# Impact of Chitooligosaccharide Conjugated Epigallocatechin Gallate and Non-Thermal High-Voltage Atmospheric Cold Plasma on *Vibrio parahaemolyticus*: An In Vitro Study and the Use in Blood Clam Meat

**DOI:** 10.3390/foods14152577

**Published:** 2025-07-23

**Authors:** Mruganxi Harshad Sharma, Avtar Singh, Ankita Singh, Soottawat Benjakul, Suriya Palamae, Ajay Mittal, Jirayu Buatong

**Affiliations:** 1 International Center of Excellence in Seafood Science and Innovation, Faculty of Agro-Industry, Prince of Songkhla University, Hat Yai 90110, Songkhla, Thailand; 6511030025@email.psu.ac.th (M.H.S.); avtar.s@psu.ac.th (A.S.); 6511030021@email.psu.ac.th (A.S.); soottawat.b@psu.ac.th (S.B.); suriya.pal@psu.ac.th (S.P.); 2 Department of Food and Nutrition, Kyung Hee University, Seoul 02447, Republic of Korea; 3 UCD Institute of Food and Health, University College Dublin, Belfield Campus, D04 V1W8 Dublin, Ireland; ajay.mittal@ucd.ie

**Keywords:** *V. parahaemolyticus*, HVACP, non-thermal technology, COS-EGCG conjugate, cell membrane damage

## Abstract

*Vibrio parahaemolyticus* is the leading cause of bacterial diarrhea in humans from shellfish consumption. In Thailand, blood clam is a popular shellfish, but homemade cooking often results in insufficient heating. Therefore, consumers may suffer from food poisoning due to *Vibrio* infection. This study aimed to determine the effect of chitooligosaccharide conjugated with epigallocatechin gallate (COS-EGCG) at different concentrations (200 and 400 ppm) combined with high-voltage atmospheric cold plasma (HVACP) on inhibiting *V. parahaemolyticus* in vitro and in challenged blood clam meat. Firstly, HVACP conditions were optimized for gas composition and treatment time (20 and 60 s); a 70% Ar and 30% O_2_ gas mixture resulted in the highest ozone formation and a treatment time of 60 s was used for further study. COS-EGCG conjugate at 400 ppm with HVACP (ACP-CE400) completely killed *V. parahaemolyticus* after incubation at 37 °C for 6 h. Furthermore, an antibacterial ability of ACP-CE400 treatment against bacterial cells was advocated due to the increased cell membrane damage, permeability, and leakage of proteins and nucleic acids. Scanning electron microscopy (SEM) showed cell elongation and pore formation, while confocal microscopy revealed disrupted biofilm formation. Additionally, the shelf life of challenged blood clam meat treated with ACP-CE400 was extended to nine days. SEM analysis revealed damaged bacterial cells on the meat surface after ACP-CE400 treatment, indicating the antibacterial activity of the combined treatment. Thus, HVACP combined with COS-EGCG conjugate, especially at a highest concentration (400 ppm), effectively inhibited microbial growth and extended the shelf life of contaminated blood clam meat.

## 1. Introduction

Bivalve mollusks, including clams, mussels, oysters, and scallops, are farmed worldwide and constitute a significant part of fishery activities. As a result, their annual growth rate is consistently strong. They are known to be filter feeders, allowing them to accumulate a large number of bacteria in their tissues, which might be potentially infectious to humans and higher vertebrates [1,2,3]. The blood clam (*Tegillarca granosa*) is a marine bivalve primarily found in intertidal mudflats along the coast of Southeast Asia and is cultivated widely due to its growing significance [4]. Blood clam is commercially cultivated in Thailand and Malaysia. The natural habitats suitable for clam cultivation are sheltered, muddy shores on the coastal mudflats between mid-neap and low spring [5]. As they are known to be bottom feeders, infectious bacteria and heavy metals from marine sediment can be absorbed by the clams and stored in their tissues, which may result in adverse effects after consumption [6]. Moreover, blood clams are often consumed raw and may be undercooked, which can lead to foodborne illness due to microbial contamination [7,8]. *V. parahaemolyticus* is a facultative, anaerobic, gram-negative, curved rod-shaped bacterium frequently found in estuarine and marine habitats [9,10]. It is one of the leading causes of foodborne gastrointestinal distress associated with the consumption of raw or undercooked seafood, characterized by symptoms such as watery diarrhea, abdominal cramps, nausea, vomiting, fever, headache, and/or bloody diarrhea, leading to potentially fatal septicemia [11]. Therefore, proper cooking or depuration of blood clams has been performed to eliminate bacteria that artificially contaminate bivalves [12]. Moreover, natural additives combined with various thermal and non-thermal techniques have been used to address this issue, ensuring the safety and preservation of seafood quality while increasing its shelf life.

Among the various non-thermal techniques being developed, high-voltage atmospheric cold plasma (HVACP) is becoming a popular method for enhancing food safety. It has emerged as a promising non-thermal, environmentally friendly technology for food processing, primarily due to its ability to inactivate microorganisms without producing toxic residues and while maintaining product quality [13,14]. During the process, an applied gas (combined or single) with a high electric field strength or energy beyond the gas ionization potential changes its state to the ionized form when excited, resulting in the production of plasma and several reactive species [15,16,17], which are known to possess excellent antimicrobial activity [18]. Various reactive species are generated during HVACP, including charged particles, electromagnetic radiation, UV radiation, energetic ions, ozone, reactive oxygen species (ROS), and reactive nitrogen species (RNS), which are known to cause enzyme inactivation, DNA cleavage, and lipid peroxidation [18,19]. Among these, ozone (O_3_) has been identified as particularly effective in suppressing bacterial growth due to its strong oxidative potential [20]. Ozone is known to have a relatively longer half-life compared to other reactive species, thus playing a key role in bacterial inactivation through its interaction with other reactive agents [21]. Apart from that, the exogenous ROS, including the highly toxic hydroxy radical (OH), damage the key cellular components such as the cell wall, cell membrane, intracellular proteins, and DNA. Moreover, the bacterial cell membrane is susceptible to these OH radicals [22]. Additionally, these ROS and RNS attack the cell wall, leading to cell rupture and peptidoglycan oxidation. Such species can also cause cell rupture through oxidative stress and damage to genetic material as well as protein denaturation [23].

Although HVACP exhibits excellent antimicrobial activity, the generation of radicals from the reactive species results in the oxidation of lipids and proteins [24]. The application of an antioxidant along with HVACP could help mitigate these negative effects. Several studies have reported the efficacy of antioxidant pretreatments in mitigating oxidative damage induced by HVACP in aquatic food products. Natural antioxidants like plant phenolic extracts, chitooligosaccharides (COS), ethanolic coconut husk extract, and chamuang leaf extract effectively scavenge reactive species generated during HVACP treatment, reducing protein and lipid oxidation and preserving the quality of treated food products.

The hydrolyzed product of chitosan, known as chitooligosaccharide (COS), has been extensively used as an antioxidant and natural antimicrobial agent, and is associated with the prevention of microbial development and lipid oxidation in fatty foods. Polyphenols, such as catechin, epicatechin, epicatechin gallate, and epigallocatechin (EGCG), possess excellent antioxidant properties. Hence, COS has been conjugated with those plant-derived polyphenols to enhance their antioxidant and antimicrobial activities [25]. Among them, COS conjugated with EGCG exhibits high antimicrobial activity, which may be enhanced when combined with HVACP, resulting in increased shelf life and reduced microbial growth [26]. Moreover, HVACP has demonstrated rapid and effective inactivation of infectious agents, particularly in the eradication of biofilm that contributes to virulence in both acute and chronic infections [27]. Clam aquaculture is increasingly challenged by the emergence of disease outbreaks and environmental contamination with an escalating prevalence of antibiotic-resistant strains. Sharma et al. [28] demonstrated the bactericidal effects of COS-EGCG conjugate against multidrug-resistant *Vibrio* strains, resulting in cell lysis. Considering the significant health risks posed by *V. parahaemolyticus* and its prevalence in filter-feeding organisms, the efficacy of either HVACP or COS-EGCG conjugate or their combination in mitigating its proliferation in clam meat requires rigorous investigation. Although COS-EGCG conjugate is employed to enhance the shelf life of mollusks such as Asian green mussel, the detailed mechanism of action of COS-EGCG conjugate and HVACP against bacterial populations, especially *V. parahaemolyticus* is still not well documented. Thus, this investigation not only explores their individual and synergistic antimicrobial effects but also addresses broader implications for consumer safety and potential applications in the food and packaging industries.

The aim of this study was to investigate the mechanism and mode of action of COS-EGCG conjugate without and with HVACP under various conditions on *V. parahaemolyticus* cells and blood clam meat inoculated with *V. parahaemolyticus* during storage at 4 °C for 9 days. Both the mode of action and the inhibition potential of the treated samples were examined to understand the mechanism of inhibition and destruction.

## 2. Materials and Methods

### 2.1. Chemicals and Microbial Media

The chitooligosaccharide (COS) was prepared using the AsA/H_2_O_2_ (Loba Chemie Pvt. Ltd., Mumbai, India) redox pair hydrolysis method as described by Mittal et al. [29]. Afterward, COS (1 g) was dissolved in 100 mL of a 0.5% (*v*/*v*) acetic acid (Sigma-Aldrich, St. Louis, MO, USA) solution and then grafted with epigallocatechin gallate (EGCG) (Xi’an Julong Bio-Tech Co., Ltd., Xi’an, China) at a concentration of 0.1% *w*/*w* relative to COS [29]. The COS-EGCG conjugate solution was dialyzed using a dialysis bag (MW cut-off: 0.5 kDa) at 4 °C for 24 h. The dialysate solution was lyophilized using a freeze dryer (Scanvac Model Coolsafe 55, Coolsafe, Lynge, Denmark). The freeze-dried powder was kept at −40 °C until use. The microbial media, including tryptic soy agar (TSA), tryptic soy broth (TSB), and thiosulfate citrate bile salt sucrose (TCBS) agar were purchased from Oxoid (Thermo Fisher Scientific, Waltham, MA, USA).

### 2.2. Preparation of Bacterial Strain

The *Vibrio parahaemolyticus* ATCC 17802 was cultured on Tryptic Soy Agar with 3% NaCl (TSA-3N) at 37 °C for 18 h. Afterward, 3–5 colonies of *V. parahaemolyticus* on TSA-3N were activated in Tryptic Soy Broth with 3% NaCl (TSB-3N) at 37 °C with shaking (180 rpm) for 4 h. Subsequently, the bacterial inoculum was prepared in 0.85% normal saline solution (NSS) to achieve a concentration of bacteria equal to 0.5 MacFarland standard (~10^8^ CFU/mL) using a densitometer (Grant bio DEN-1, Grant Instruments Ltd., Cambridge, UK).

### 2.3. High Voltage Cold Atmospheric Plasma (HVACP) in the Package Treatment

The system of HVACP operates as a surface dielectric barrier discharge (SDBD) with a high-voltage transformer (input voltage of 115 V at a frequency of 50 Hz) and a variable voltage transformer (VARIAC) with a range of 0–120 V. A mixed gas of argon (Ar) and oxygen (O_2_) with different ratios was added to the polypropylene box (115 × 82 × 104 cm) and sealed using a modified atmosphere packaging (MAP) machine model DMP-430 (Zhejiang Ruibao Packaging Machinery Co., Ltd., Ruian, China). There were 3 different ratios of argon (Ar) and oxygen (O_2_), including 70% Ar + 30% O_2_, 80% Ar + 20% O_2_, and 90% Ar + 10% O_2_. Furthermore, all these boxes filled with mixed gases of varying ratios were subjected to HVACP at 30 k peak-to-peak voltage (Vpp) for 2 different treatment times (20 s and 60 s). All experiments were carried out in duplicate. Ozone concentrations generated were measured using GASTEC gas tube detectors (Product # 18 mol L^−1^, Gastec Corporation, Kanagawa, Japan) immediately after treatment.

The gas mixture ratio of 70% Ar + 30% O_2_ showed the highest level of ozone (O_3_) gas at 60 s of HVACP treatment time was selected for further study.

### 2.4. Effect of Combined Treatment of COS-EGCG Conjugate and HVACP on V. parahaemolyticus

#### 2.4.1. Time-Kill Analysis

*V. parahaemolyticus* cells were mixed with COS-EGCG conjugate (200 and 400 ppm) and then treated with HVACP. In brief, *V. parahaemolyticus* was cultured in TSB-3N medium at 37 °C for 24 h. The inoculum of *V. parahaemolyticus* (~10^6^ CFU/mL) in TSB-3N medium was prepared [30]. The inoculum of *V. parahaemolyticus* was mixed with COS-EGCG conjugate in equal volume to obtain the final concentration of COS-EGCG conjugate at 200 and 400 ppm [31]. The inoculum of *V. parahaemolyticus* mixed with TSB-3N medium was considered as the control. The treatments of bacteria treated with COS-EGCG conjugate with and without HVACP were prepared (Table 1). All treated packages with HVACP were incubated at 37 °C for 48 h and the number of bacteria was counted at 0, 3, 6, 9, 12, 15, 24, and 48 h using serial dilution with NSS, and the viable bacteria were counted on the TCBS agar by the plate count method. Time-kill analysis was performed to monitor the antibacterial activity of COS-EGCG conjugate against *V. parahaemolyticus* under different treatment conditions.

#### 2.4.2. Cell Membrane Permeability

The effects of different treatments on the permeability of *V. parahaemolyticus* cell membranes were determined using confocal laser scanning microscopy (CLSM) (TI-EAI, NIKON, Tokyo, Japan). After being treated by COS-EGCG conjugate (200 and 400 ppm) with and without HVACP, 10 μL of cell suspension was added to 990 μL of sterilized saline water containing 3.0 μL of propidium iodide (PI, Invitrogen, Carlsbad, CA, USA) and incubated in the dark at room temperature for 20 min [32]. The red fluorescence of dead *V. parahaemolyticus* cells, which had lost their ability to permeabilize cell membranes, was observed using PI staining.

#### 2.4.3. Protein and Nucleic Acid Leakage

Protein, peptide, and nucleic acid remaining in the *V. parahaemolyticus* cells were determined by FTIR analysis [33]. Prepared bacterial suspensions treated with COS-EGCG conjugate at varying concentrations (200 and 400 ppm) with and without HVACP (30 kVpp, 60 s) were examined after incubation at 37 °C for 24 h. The bacterial cells were then collected by centrifugation at 3773.25× *g* for 10 min followed by washing three times with 0.1 M phosphate buffer solution (PBS), pH 7.2. The pelleted cells were freeze-dried using a freeze dryer (Scanvac Model Coolsafe 55, Coolsafe, Lynge, Denmark) for 48 h and then tested with a Fourier infrared spectrometer (Bruker INVENIO-S FTIR spectrometer, Bruker Co., Ettlingen, Germany). The proteins, peptides, and nucleic acids remaining in the bacterial cells were measured by observing the peaks in their respective regions.

#### 2.4.4. Biofilm Formation

The effect of the COS-EGCG conjugate with and without HVACP on the biofilm formation ability of *V. parahaemolyticus* was evaluated by using a CLSM. COS-EGCG conjugate solution was prepared in 24-well plates at different concentrations in triplicate. An overnight culture of *V. parahaemolyticus* in TSB-3N was used to prepare an inoculum (~10^6^ CFU/mL), and then the inoculum was added into 24-well plates to obtain the final concentration of COS-EGCG conjugate at 200 and 400 ppm. The 24-well plates containing COS-EGCG conjugate with bacteria were treated with HVACP (30 kVpp, 60 s), and the 24-well plates were incubated at 37 °C for 48 h to produce the biofilm. Thereafter, the wells were washed gently, twice with PBS to remove planktonic cells. The staining dye was prepared by adding 10 µL of SYBR^®^ Green I Nucleic Acid Gel Stain (catalog number S7563) (Invitrogen, Carlsbad, CA, USA) into 1.0 mL of sterile dH_2_O and vortexing thoroughly. The stain (10 mL) was then added to each well and mixed thoroughly. The plate was incubated at room temperature in the dark for 15 min. Thereafter, the effect on the biofilm was studied using a CLSM (LSM710, Carl Zeiss AG, Oberkochen, Germany) with an excitation wavelength of 497 nm and an emission wavelength of 520 nm [34].

#### 2.4.5. Structure and Morphology of Cells

The *V. parahaemolyticus* cells treated with HVACP (30 kVpp for 60 s), COS-EGCG conjugate (400 ppm), and HVACP (30 kVpp for 60 s) combined with COS-EGCG conjugate (400 ppm) were chosen to study changes in the morphology of cells using scanning electron microscopy (SEM) and transmission electron microscopy (TEM). SEM was performed using an FEI Quanta 400 scanning electron microscope (FEI, Brno, Czech Republic). The cell suspension for each treatment after being incubated at 37 °C for 24 h was prepared. The control sample comprised bacterial cells that underwent no treatment. The treated samples as well as the control were further centrifuged at 3000× *g* for 5 min, and the cell pellet was then resuspended in 0.1 M phosphate buffer (pH 7.2). Furthermore, fixation and dehydration were performed as described by Buatong et al. [35]. Similarly, the changes in structure and composition of *V. parahaemolyticus* cells were also determined. After treatment and incubation, the *V. parahaemolyticus* cells were visualized using a transmission electron microscope (TEM, JEOL Ltd., Tokyo, Japan) [36]. TEM observed the internal structures within the bacterial cells, such as organelles (vacuoles, nuclei, mitochondria), and determined the detailed ultrastructure of the cell wall and membrane.

### 2.5. Effect of Combined Treatment of COS-EGCG Conjugate and HVACP on V. parahaemolyticus Inoculated on Shucked Blood Clam Meat

#### 2.5.1. Preparation of Blood Clam Meat

**Figure 1:**
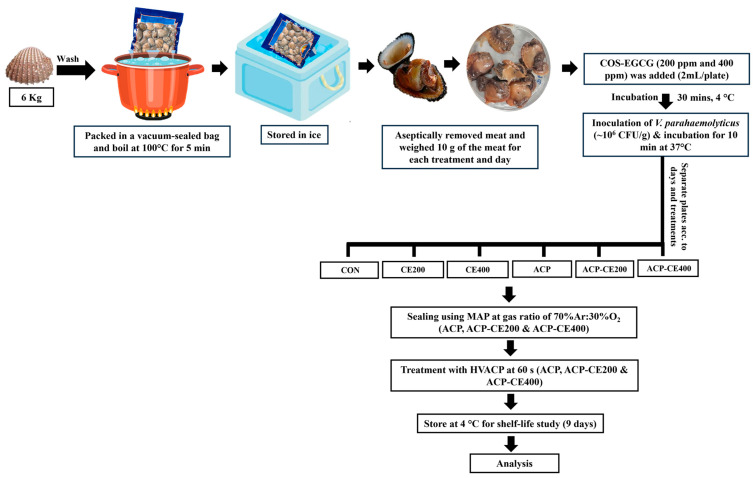
Experimental outline for preparation of challenged blood clam meat.

Firstly, 6 kg of fresh live blood clams (*T. granosa*), each weighing 10–12 g, were bought from a local market in Hat Yai, Thailand, and transported to the laboratory in an ice box. After arriving at the laboratory, the blood clams were washed with sterilized distilled water to remove mud, sand, and other foreign particles. For shucking, blood clams were vacuum sealed in a double-layered bag composed of linear low-density polyethylene (LLDPE) and polyamide and then boiled at 100 °C for 5 min. Thereafter, the blood clams were stored in an ice box to cool down, and then shucking was performed under an aseptic condition. The meat of the blood clam was then divided into 6 different parts (10 g each). For COS-EGCG treatment, the conjugated powder was dissolved in the minimum amount of water, and the final concentrations were maintained at 200 and 400 ppm. Then, the conjugate solution (2 mL) was poured onto the 10 g of meat placed in a Petri plate and then appropriately mixed. Thereafter, each plate was incubated at 4 °C for 30 min to allow COS-EGCG conjugate to distribute or adhere to the surface of the blood clam meat. After incubation, 100 µL of *V. parahaemolyticus* inoculum (~10^8^ CFU/mL) was inoculated onto the meat (10 g) present in the Petri plate. Afterwards, the plates were incubated at 37 °C for 10 min. The final concentration of *V. parahaemolyticus* was ~10^6^ CFU/g. Finally, those treated plates were placed into a polypropylene box and filled with a selected gas ratio, followed by sealing using an MAP machine. Then, the boxes containing 200 or 400 ppm conjugate were treated both with and without HVACP at selected conditions. The samples without any treatment and with only HVACP were defined as CON and ACP, respectively. Samples added with 200 and 400 ppm conjugates were named as CE200 and CE400, respectively. Samples added with 200 and 400 ppm conjugate, treated with HVACP, were named ACP-CE200 and ACP-CE400, respectively. All the treatments were performed in duplicate. The whole experiment is outlined in Figure 1. The boxes were then stored at 4 °C for 9 days and analyzed on days 0, 3, 6, and 9.

#### 2.5.2. *V. parahaemolyticus* Count

For *Vibrio* count, the blood clam meat (10 g) was homogenized with NSS (90 mL) using a stomacher (Mode l400, Seward Ltd., West Sussex, UK) for 1 min at 230 rpm. The homogenate was further serially diluted 10 times in 0.85 % NSS. Each suspension was then spread on TCBS agar and incubated at 37 °C for 24 h. The bluish-green colonies developed on TCBS agar plates representing *V. parahaemolyticus* were counted.

#### 2.5.3. Scanning Electron Microscopy (SEM)

The treatment showing the lowest number of *V. parahaemolyticus* was subjected to SEM analysis in comparison to the CON on day 9 of storage. The whole blood clam meat was cut into small pieces (1 cm × 1 cm) in duplicate and placed in a 6-well plate. Samples were fixed with glutaraldehyde (2.5%) for 2 h at room temperature. Thereafter, the fixed samples were washed 3 times with 0.1 M phosphate buffer (pH 7.2). The dehydration of the fixed samples was then carried out using a series of ethanol concentrations from 50% to 99.9%, followed by drying to the critical point. It was sputtered for 1 min with a gold coat and visualized using an FEI Quanta 400 scanning electron microscope (FEI Czech Republic, Brno, Czech Republic) [35].

### 2.6. Statistical Analysis

Completely randomized design (CRD) was used to carry out the study. GraphPad Prism version 8 (GraphPad Software, Boston, MA, USA) was used to perform an analysis of variance (ANOVA) with Tukey’s test for the mean comparison. A *p* < 0.05 indicated statistical significance.

## 3. Results and Discussion

### 3.1. Effect of Argon-Oxygen Gas Composition on Ozone Generation

The bactericidal effect of HVACP is mainly due to the production of ROS and reactive nitrogen species (RNS) in the presence of Ar [37]. The efficacy of HVACP increases significantly in the presence of ozone (O_3_) when air or oxygen acts as a feeding gas [38]. Ozone concentrations were measured immediately after plasma treatment using GASTEC gas tubes. The results demonstrated that the gas composition of 70% Ar + 30% O_2_ produced the highest concentration of ozone when treated for 60 s (Table 2). The lowest Ar percentages and highest O_2_ contents in the mixture resulted in the maximum ozone synthesis, demonstrating a synergistic effect between oxygen and plasma discharge in producing ROS, such as O_3_ [39]. According to Olatunde et al. [40], HVCAP generated using Ar at ambient temperature demonstrated significant antibacterial efficacy, but its practical application remained constrained due to the lower generation of ozone among its reactive species. To overcome this limitation, it is imperative to optimize the controlled introduction of oxygen, thereby facilitating ozone generation and enhancing the overall antimicrobial potential of the Ar/O_2_-based HVCAP system. Furthermore, packages treated with 30 kVpp for 20 s resulted in a lower ozone concentration, highlighting the significance of both exposure time and the voltage in generating ozone. Hemmati et al. [41] found that increasing the duration of cold plasma treatment results in higher antibacterial activity for a variety of products, including red pepper powder, turmeric powder, and snacks.

Moreover, the inactivation mechanism of ozone involves progressive oxidation of cellular constituents. The antimicrobial activity of ozone is attributed to its oxidant potential, which causes visible damage to the fatty acids and proteins present in the cellular components of pathogenic bacteria. This oxidation potential and antimicrobial effect make it a promising agent in food processing [42]. Ozone is approved as an antibacterial agent for food processing, storage, treatment, and is classified as GRAS (Generally Recognized as Safe) by the US Food and Drug Administration (FDA) [43]. Thus, the condition with 70% Ar + 30% O_2_ at 30 kVpp for 60 s was considered for further studies.

### 3.2. Effects of COS-EGCG Conjugate Without and with HVACP Treatment at Selected Conditions on V. parahaemolyticus Cells

#### 3.2.1. Time-Killing Analysis

**Figure 2:**
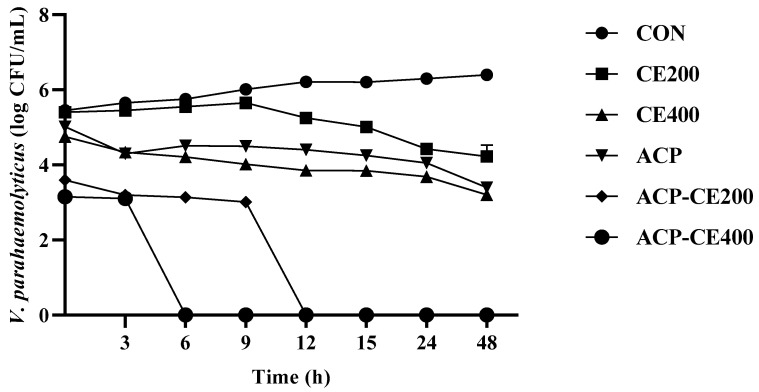
Antibacterial effect of the COS-EGCG conjugate combined with and without HVACP treatment against *V. parahaemolyticus* after every 3 h for 48 h. CON: control without any treatment; CE200: COS-EGCG conjugate at 200 ppm; CE400: COS-EGCG conjugate at 400 ppm; ACP: high-voltage atmospheric cold plasma (HVACP); ACP-CE200: HVACP combined with COS-EGCG conjugate at 200 ppm and ACP-CE400: HVACP combined with COS-EGCG conjugate at 400 ppm. HVACP treatment was performed using a gas mixture ratio of 70% Ar: 30% O_2_ for 60 s.

*V. parahaemolyticus* showed exponential growth in the absence of COS-EGCG conjugate and HVACP (CON), reaching 6.4 log CFU/mL in 24 h (Figure 2). Treatment CE200 demonstrated a limited bacteriostatic effect until 9 h with a gradual decline in *V. parahaemolyticus* count thereafter. When the concentration of COS-EGCG conjugate was increased to 400 ppm, the time required to achieve a 1.3 log CFU/mL reduction in cell viability was reduced to 6 h. The inhibition of bacterial growth may be attributed to the chelation of essential micronutrients, such as ferric (Fe^3+^) and ferrous (Fe^2+^), by EGCG/COS [44] or the electrostatic interactions between positively charged amino group (NH^3+^) of COS and the negatively charged bacterial membrane, which alter bacterial cell wall integrity resulting in microbial reduction. A similar trend was observed after 3 h in the ACP treatments, showing a bacterial reduction to 1 log CFU/mL, respectively, in comparison to the CON.

According to Chen et al. [45], total viable counts (TVC) of samples treated with atmospheric cold plasma were significantly lower than those of the untreated control, indicating the high decontamination efficacy of the treatment. They observed that the atmospheric cold plasma treatment reduced the initial TVC from 3.01 log CFU/g to 1.58 log CFU/g, underscoring the sustained antimicrobial effect of cold plasma during cold storage. The combination of HVACP with COS-EGCG conjugate markedly improved the bactericidal activity of the conjugate. Compared to other treatments, ACP-CE400 resulted in the complete killing of *V. parahaemolyticus* within 6 h, whereas ACP-CE200 treatment took 12 h to achieve a similar impact. The results also confirmed that the increasing concentration of COS-EGCG conjugates enhances its antibacterial ability. In general, the antibacterial activity of HVACP is mainly due to the generation of free radicals, ROS, including O_3_, H_2_O_2_ and NO_x_, which have the potential to damage and pass through cell membranes into the cytoplasm, targeting microbial DNA, and ultimately resulting in cell death [46,47]. However, O_3_ gas has a short lifetime and then quickly decomposes back into O_2_. The O_3_ gas produced by the HVACP (80 kVRMS) using a mixture of argon and oxygen at a ratio of 90:10 and 80:20 for 1, 1.5, and 2.5 min decomposed after treatment for 24 h has been reported [40]. Therefore, O_3_ gas exhibits high reactivity toward bacterial cells during the initial stages of treatment. Its rapid oxidative action contributes to early cell membrane damage, while COS-EGCG conjugate provides a sustained antimicrobial effect through a synergistic mechanism, ultimately leading to bacterial inactivation. This synergistic effect may imply that HVACP enhances the antimicrobial potential of COS-EGCG conjugate by inducing bacterial membrane disintegration, oxidative damage, and increased incorporation of the conjugate.

#### 3.2.2. Effects of COS-EGCG Conjugate Without and with HVACP Treatment on Cell Membrane Permeability of *V. parahaemolyticus*

**Figure 3:**
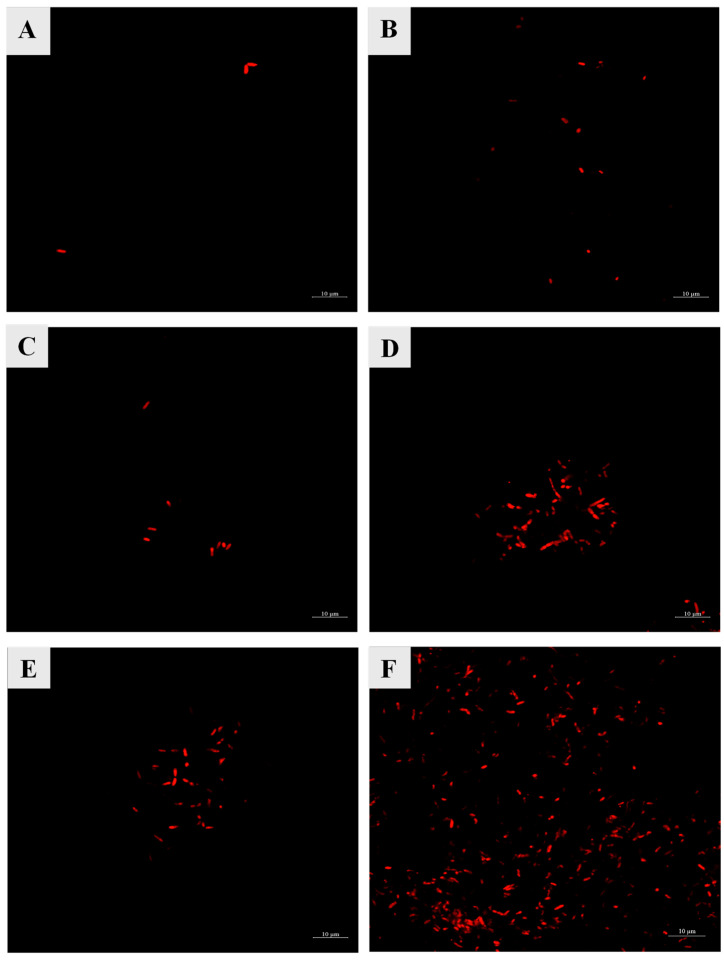
Cell membrane permeability of *V. parahaemolyticus* cells treated with COS-EGCG conjugate in combination with and without HVACP. (**A**) CON, (**B**) ACP, (**C**) CE200, (**D**) ACP-CE200, (**E**) CE400, and (**F**) ACP-CE400. For abbreviations see Figure 2 caption.

Cell membrane damage was indicated by an increase in red fluorescence signal in the treatments ACP-CE400 followed by ACP-CE200 and CE400, respectively (Figure 3). The red fluorescence signal of CE200 and ACP treatments showed no difference. On the other hand, the bacterial cells in CON exhibited the lowest fluorescence signal, indicating that their cell membranes were intact. Fluorescence imaging revealed the effect of HVACP and COS-EGCG conjugate treatment on bacterial membrane integrity. A red fluorescence signal is created when PI attaches to DNA in cells with damaged membranes [48]. PI is supposed to only enter cells with damaged cell membranes and is typically excluded from viable cells. It is commonly used to detect dead cells in a population and as a counterstain in multicolor fluorescence techniques [49].

HVACP has the potential to damage Gram-negative bacterial cells by disrupting the cell membrane, which induces leakage of intracellular materials (e.g., DNA) and perforation due to an etching effect. The charged ions or discharge gases break the chemical bonds, particularly those of hydrocarbons, leading to the formation of pores in the cell membrane. This is driven by the accumulation of charged particles on the membrane, where electrostatic stress overcomes the tensile strength, resulting in the rupture of the bacterial cell [50]. Moreover, the outer membrane of Gram-negative bacteria contains lipopolysaccharides, and the cell wall consists of a thin peptidoglycan layer. The ROS generated during HVACP can interact with these structural components in the bacterial cell, resulting in the degradation of molecules through the breakage of C–O, C–N, and C–C bonds [51]. These findings demonstrate that the combination of HVACP and COS-EGCG conjugate leads to widespread membrane permeability, potentially enhancing antibacterial effectiveness. On the other hand, *V. parahaemolyticus* cells treated with COS-EGCG conjugate alone showed a lower red fluorescence signal. However, treatment CE400 (Figure 3E) showed a higher fluorescence signal in comparison to HVACP alone (Figure 3B), indicating the effect of COS-EGCG conjugate on cell membrane disruption. The results were also in agreement with the lower time and bacterial counts associated with the treatments, especially the combined treatments (Figure 2). The higher concentrations of EGCG intensify its destructive impact on bacterial membranes, increasing permeability. This enhanced permeability facilitates the entry of EGCG into the cells, disrupting normal physiological and metabolic processes [52]. The ROS generated during HVCAP exposure accelerate the oxidation of cellular components, including lipids, amino acids, and nucleic acids [53]. These reactive species can also induce modifications in microbial DNA, ultimately leading to cell inactivation. The antibacterial activity of HVACP is attributed to the action of free radicals and the accumulation of charged particles, which leads to the rupture of the bacterial cell membrane [40].

#### 3.2.3. Protein and Nucleic Acid Leakage of V. parahaemolyticus Cells Treated With COS-EGCG Conjugate in Combination With and Without HVACP

**Figure 4:**
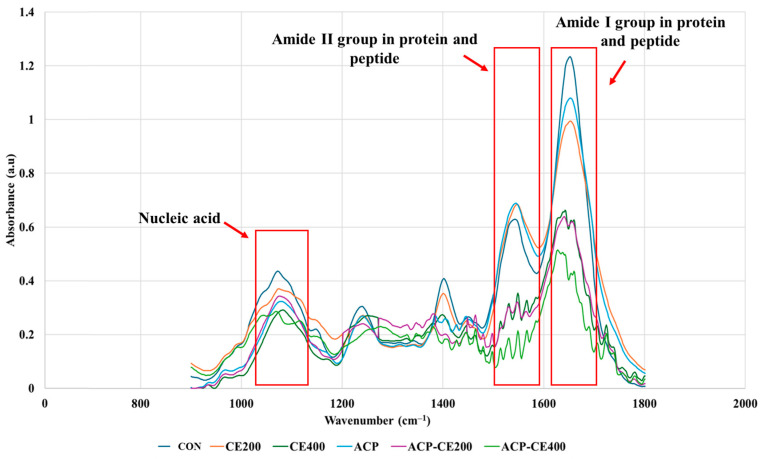
FTIR spectra of freeze-dried *V. parahaemolyticus* cells after treatment with COS-EGCG conjugate in combination with and without HVACP. For abbreviations see Figure 2 caption.

FTIR spectral analysis revealed distinct alterations in the protein and nucleic acid structures of *V. parahaemolyticus* cells following HVACP and COS-EGCG treatment (Figure 4). The peaks within the wavenumber range of 1800–1500 cm^−1^ are influenced by amide I and amide II groups present in proteins and peptides. The amide I band corresponds predominantly to the C=O stretching vibrations within the peptide bond. In contrast, the amide II band primarily arises from N–H bending vibrations, along with contributions from C–N stretching [54]. These regions exhibit strong peaks that provide detailed information about protein structures, as well as the vibrations of ester functional groups in lipids and nucleic acids. Meanwhile, the 900–1200 cm^−1^ range is dominated by symmetric stretching vibrations of PO_2_^−^ groups in nucleic acids and includes signals related to carbohydrates, polysaccharides, and nucleic acids [33].

Untreated samples exhibited the highest peaks of intensity within both spectral windows, indicating a high concentration of intact proteins and nucleic acids within the bacterial cell. On the contrary, ACP-CE400 exhibited the weakest intensity peaks, indicating the leakage of proteins and nucleic acids from the cells, followed by ACP-CE200 and CE400. The HVACP induces the degradation of cellular proteins, with larger proteins (50–90 kDa) being specifically targeted for degradation [55]. This degradation mechanism might be due to reactive compounds in the plasma disrupting hydrogen, sulfide, and peptide bonds. Such alterations lead to changes in the primary, secondary, and tertiary structures of proteins, ultimately reducing enzymatic activity and cellular mechanisms. Buatong et al. [35] also observed protein leakage from *Listeria monocytogenes* cells treated with COS-EGCG conjugate, which was primarily attributed to the disruption of the cell membrane. The disruption in bacterial nucleic acid is primarily through the action of ROS and RNS released during HVACP, which targets the backbone of DNA leading to the extraction of hydrogen atoms and formation of radicals that break N-glycosidic bonds, resulting in the release of nitrogenous bases and fragmentation of the deoxyribonucleotide chain. Moreover, UV photons generated by HVACP contribute to nucleic acid damage by forming thymine dimers, which introduce structural abnormalities in DNA and impair bacterial replication [50]. CE400 and ACP-CE400 depicted lower peaks suggesting a relatively higher effect on protein and nucleic acid denaturation. EGCG exhibits the potential to inhibit the production of nucleic acids (DNA and RNA) and modify the DNA methylation pattern by interfering with the metabolism of folic acid in bacterial cells [56]. This corresponds to a previously reported study on *Shewanella putrefaciens* treated with EGCG [57]. This reduction in proteins and nucleic acids highlights the combined antimicrobial activity of COS-EGCG conjugate and HVACP, which disrupts cellular macromolecules.

#### 3.2.4. Inhibition of Biofilm Formation

**Figure 5:**
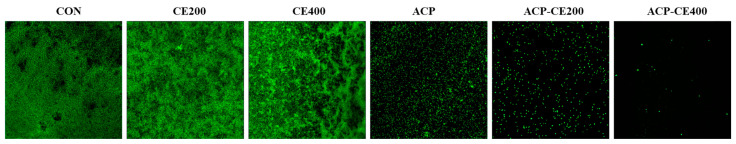
CLSM analysis of biofilm formation by *V. parahaemolyticus* cells treated with COS-EGCG conjugate in combination with and without HVACP. For abbreviations see Figure 2 caption.

The biofilm of *V. parahaemolyticus* was allowed to form at 37 °C. In the present study, the untreated control samples (CON) exhibited thick biofilm matrices at 37 °C, indicating a high number of viable cells as indicated by the higher green fluorescence (Figure 5). The CLSM examination using SYBR Green I staining demonstrated biofilm cell viability and structural integrity. SYBR Green I dye, which is primarily used in the staining of double-stranded DNA in molecular biology, can also be used in the bacterial viability assessment by staining the live or viable cells with an intact membrane that appears green under fluorescence [34]. Biofilms are complex microbial communities embedded in hydrated extracellular polymeric substances (EPS), which are composed of polysaccharides, proteins, phospholipids, and nucleic acids. The heterogeneous nature of these structures renders bacterial biofilms more resistant compared to their planktonic counterparts allowing them to withstand various environmental stresses and other adverse conditions that hinder bacterial growth [58].

Treatments with CE400 and ACP-CE400 revealed structural biofilm changes, characterized by low cell density and a turbid appearance, demonstrating efficient biofilm inhibition. The cumulative action of ACP-CE400 caused a drastic breakdown of the biofilm, accompanied by a low intensity of the biofilm layer. The fluorescence intensity followed a decreasing trend: CON > CE200 > CE400 > ACP > ACP-CE200 > ACP-CE400 (Figure 5). The biofilm formation inhibition in samples treated with COS-EGCG conjugate alone at 200 and 400 ppm did not yield effective results; however, the inhibition was higher than that of the CON. This might be due to the activity of COS-EGCG conjugate at low concentrations in preventing biofilm formation without inhibiting bacterial growth, thereby giving the bacterial cell an opportunity for biofilm recolonization [59]. The reactive species generated during the HVACP treatment are potentially able to penetrate and disrupt the biofilm structure, targeting the bacteria and subsequently leading to cell death [60]. These reactive species eliminate the bacterial population and facilitate the breakdown of the biofilm matrix, preventing recolonization by new biofilms. The findings demonstrate the efficient potential of combining HVACP and COS-EGCG conjugate as a feasible strategy for preventing biofilm development, improving food safety, and reducing the risks of microbial contamination in food processing environments.

#### 3.2.5. Structural and Morphological Changes Observed by SEM and TEM

**Figure 6:**
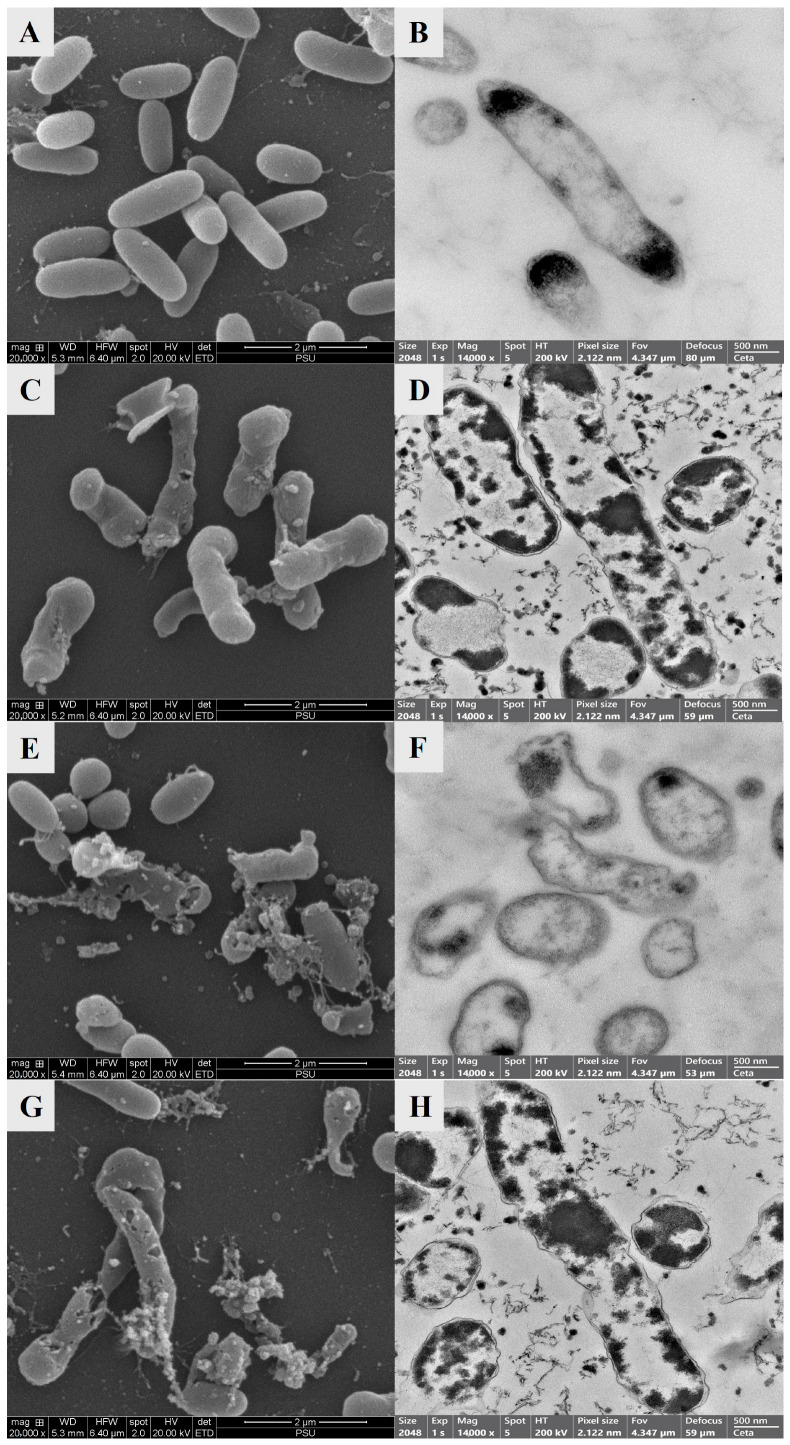
Scanning electron microscopy (SEM) (**A**,**C**,**E**,**G**) and transmission electron microscopy (TEM) (**B**,**D**,**F**,**H**) analyses of *V. parahaemolyticus* cells treated with COS-EGCG conjugate in combination with and without HVACP. (**A**,**B**) CON, (**C**,**D**) CE400, (**E**,**F**) ACP, and (**G**,**H**) ACP-CE400. For abbreviations see Figure 2 caption.

Significant morphological and structural variations were found between several treatment groups of *V. parahaemolyticus* cells using SEM and TEM. Untreated *V. parahaemolyticus* cells (Figure 6A,B) exhibited a smooth, intact outer membrane along with well-preserved cellular components that maintained structural integrity and cell viability. In contrast, treatment CE400 (Figure 6C,D) resulted in cell elongation and extensive cell wall disruption, supporting the antimicrobial activity of the conjugate.

Cell elongation might be due to suppression of cell division protein known as filamentation temperature-sensitive mutant Z (FtsZ). FtsZ is responsible for the formation of the Z-ring at the mid-cell facilitating cell division. EGCG exhibits the potential to interfere with the polymerization of FtsZ, resulting in the cell forming a septum, hindering normal cell division. This consequently leads to the elongation of bacterial cells as they continue growing without division [61]. Additionally, cells treated solely with HVACP displayed notable deformation such as the formation of a pore on the cell surface (Figure 6E,F). This might be due to the generation of charged ions, which accumulated, leading to cell rupture [62]. The data were also supported by the higher protein leakage (Figure 5). Treatment with ACP-CE400 demonstrated a synergistic effect, as evidenced by both cell elongation and pore formation (Figure 6G,H). The metastable ions produced during HVACP interact with water molecules to generate OH radicals, which target the outer cell layer, disrupting the DNA structure. This implies that by rupturing the cell membrane, HVACP renders the bacteria more susceptible to COS-EGCG conjugate, allowing the conjugate to penetrate the bacterial cytoplasm more deeply. These pores most likely allowed intracellular components to escape leading to the inhibition of bacterial growth. According to Ravash et al. [63], the reactive species produced during HVACP result in lipid peroxidation, protein oxidation, and chemical bond dissociation, which induce membrane erosion, perforation, and rupture leading to cell leakage and impaired membrane function. Furthermore, proteins, enzymes, and genetic material (DNA and RNA) are harmed by plasma-induced oxidative stress, which hinders cell division and replication. Higher plasma electric fields cause membrane tension, electroporation, and intracellular oxidative stress resulting in apoptosis, thus lowering cell viability and proliferation.

### 3.3. Impact of COS-EGCG Conjugate in Combination with HVACP on V. parahaemolyticus Cells Inoculated on Blood Clam Meat

**Figure 7:**
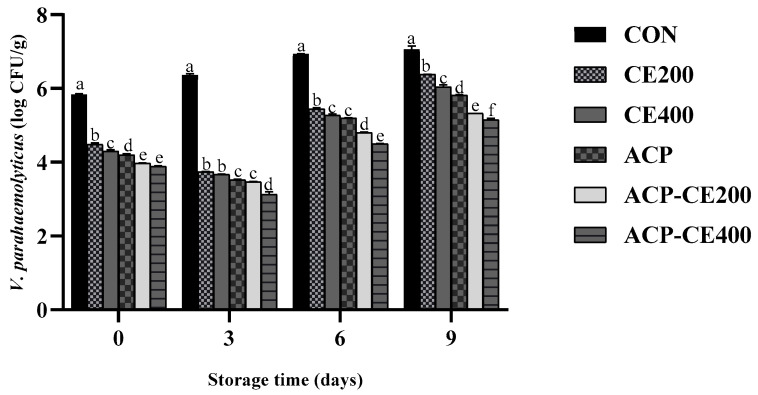
Changes in *V. parahaemolyticus* count in inoculated blood clam meat treated with COS-EGCG conjugate in combination with and without HVACP during storage of 9 days at 4 °C. The bars represent the standard deviation (*n* = 3). Different lowercase letters on the bars within the same storage time indicate significant differences (*p* < 0.05). For abbreviations see Figure 2 caption.

The study revealed the antimicrobial effect of COS-EGCG conjugate combined with HVACP in inhibiting *V. parahaemolyticus* in blood clam meat. On day 0, the untreated sample (CON) showed the highest *V. parahaemolyticus* count (VC) with 5.83 log CFU/g (Figure 7). On the other hand, treatments CE200 and CE400 demonstrated significantly lower VC with 4.49 and 4.30 log CFU/g, respectively (*p* < 0.05). Whereas the lowest VC was obtained in treatments ACP-CE400 and ACP-CE200 with 3.89 and 3.97 log CFU/g, respectively, followed by ACP (4.2 log CFU/g) (*p* < 0.05).

**Figure 8:**
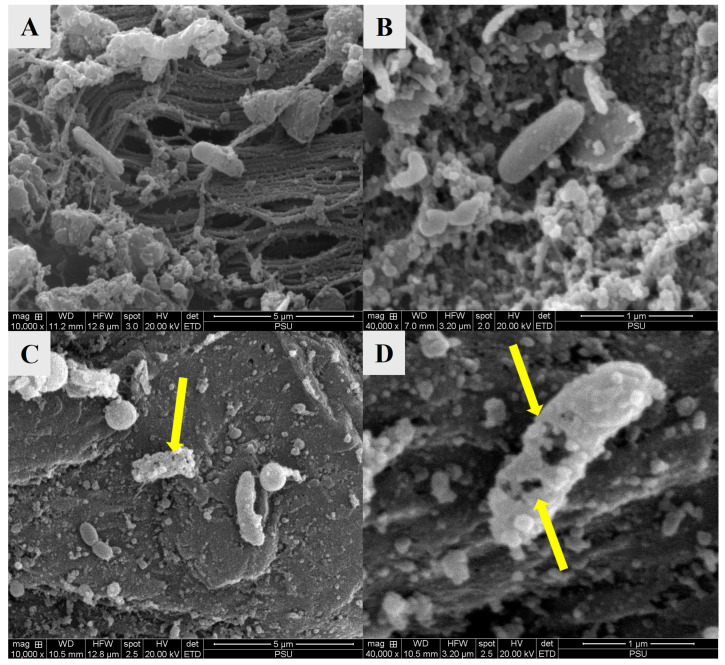
Morphological changes of *V. parahaemolyticus* cells adhered to the surface of blood clam meat in control group (**A**,**B**) and treated with COS-EGCG conjugate 400 ppm in combination with HVACP (**C**,**D**) on day nine of the shelf-life study. The yellow arrows indicated the pores on *V. parahaemolyticus* cells.

Over the storage period, a significant increase in VC was observed, and the treatments showed a noteworthy reduction in bacterial growth compared to the CON, where CON showed increasing VC and attained the highest value on day 9. On the other hand, the lowest VC was observed on day 3 for the treated sample, which may be attributed to the initial growth-suppressing action of COS-EGCG conjugate and HVACP treatment. In contrast to day 0, the samples treated solely with ACP yielded higher VC than the CE200 and CE400 treatments (*p* < 0.05). The COS-EGCG conjugate was found to exhibit potent antibacterial activity, resulting from its action on the bacterial membrane, enzyme inhibition and induction of oxidative stress [64]. However, by day 9, the VC was significantly increased in all sample groups (*p* < 0.05), underscoring the limitations of treatment under prolonged storage conditions. These undesirable changes might be due to lipid oxidation and phenolic degradation. The ROS are generated in larger amounts when oxygen is applied as a constituent gas in HVACP treatment leading to deteriorating food quality, which is caused by the lipid oxidation initiated by oxygen-mediated treatment [65]. Moreover, Han et al. [51] reported that the inactivation of Gram-negative bacteria by HVACP is primarily attributed to oxidative damage induced by ROS leading to membrane disruption, leakage of intracellular contents, and eventual cell lysis. However, for a shorter period of storage, these ROS, RNS, positively and negatively charged species, short-lived radicals and radiation either act directly or indirectly with the treated food matrix, thereby decontaminating and preserving their attributes similar to freshness [66]. Furthermore, when the microstructure of bacteria adhered to the surface of blood clam meat was imaged using SEM for the selected sample (ACP-CE400) in comparison to the CON. The cells on the CON showed intact rod-shaped cells with a smooth outer cell membrane (Figure 8A,B) and there was no disruption in bacterial structure. On the contrary, treatment ACP-CE400 showed evident changes in morphology (Figure 8C,D), where the cell wall of the treated sample was observed to be damaged with pores and a rough surface.

Moreover, the shape of the cells was disoriented, which suggests that COS-EGCG conjugate and HVACP were effective. The result was also supported by the in vitro analysis of bacterial cells (Figure 5). This may be due to the loose, cross-linked cell wall structure of peptidoglycan, which leads to oxidative damage and cell membrane rupture, ultimately resulting in bacterial inactivation [67]. Additionally, the disrupted cell wall allowed the penetration of COS-EGCG conjugate, which may interfere with protein synthesis and RNA/DNA processing. Buatong et al. [35] reported that COS-EGCG conjugate exhibits the potential to disrupt membrane integrity by altering DNA and RNA, leading to reduced growth of *L. monocytogenes*. Similar results were noticed against *Pseudomonas* spp. when treated with COS from squid pen at different concentrations in combination with HVACP [26]. Thus, the combination of COS-EGCG conjugate with HVACP caused a deformed and distorted cell structure, exhibiting pores that resulted in the leakage of nutrients and ultimately led to cell lysis.

## 4. Conclusions

The impact of different gas ratios on ozone generation using HVACP at various treatment times revealed that a gas composition of 70% Ar + 30% O_2_ and a 60 s treatment yielded the highest ozone production. In vitro analysis of COS-EGCG conjugate, especially at the highest concentration (400 ppm), suggested the disintegration of the cell membrane, leading to the leakage of proteins and nucleic acids from *V. parahaemolyticus* cells, especially when combined with HVACP (ACP-CE400). Moreover, the inhibition of biofilm formation of *V. parahaemolyticus* by the combined technique was studied using live cells. ACP-CE400 demonstrated the highest inhibition of biofilm, accompanied by the fewest alive cells. The antibacterial efficacy of the combined treatment was also supported by the distorted bacterial shape as visualized by SEM and TEM. Similarly, ACP-CE400 treatment successfully inhibited the growth of *V. parahaemolyticus* inoculated on the blood clam meat. The shelf-life of blood clam meat incorporated with ACP-CE400 was extended to nine days. The combined effect was not immediately visible, but it resulted in very low bacterial growth on day three, which subsequently increased by day six. Scanning electron microscopy revealed that *Vibrio* cells on the surface of ACP-CE400 treatment were predominantly deceased or exhibited extensive disintegration by day nine, indicating the effectiveness of the treatment in compromising cellular integrity. Hence, cultured blood clam meat infected with *V. parahaemolyticus* can be controlled by the application of HVACP and COS-EGCG conjugate, ensuring consumer safety.

## Figures and Tables

**Table 1:** The details of treatments in combination of COS-EGCG conjugate and HVACP.

Treatments	Details of the Treatments
Control	bacterial inoculum without any treatment
CE200	bacterial inoculum treated with 200 ppm of COS-EGCG conjugate
CE400	bacterial inoculum treated with 400 ppm of COS-EGCG conjugate
ACP	bacterial inoculum treated with HVACP using gas mixture ratio of 70% Ar:30% O_2_ for 60 s
ACP-CE200	bacterial inoculum treated with 200 ppm of COS-EGCG conjugate combined with HVACP using gas mixture ratio of 70% Ar:30% O_2_ for 60 s
ACP-CE400	bacterial inoculum treated with 400 ppm of COS-EGCG conjugate combined with HVACP using gas mixture ratio of 70% Ar:30% O_2_ for 60 s

**Table 2:** Ozone concentration in the package contained different gas compositions after HVACP process at 30 kVpp for different times.

Gas Composition		Ozone Concentration (ppm)		
		70% Ar + 30% O_2_	80% Ar + 20% O_2_	90% Ar + 10% O_2_
Time	20 s	200	80	100
	60 s	240	20	20

## Data Availability

The original contributions presented in the study are included in the article, further inquiries can be directed to the corresponding author.
